# Goitre Prevalence and Urinary Iodine Concentration in School-Aged Children in the Ashanti Region of Ghana

**DOI:** 10.1155/2020/3759786

**Published:** 2020-03-23

**Authors:** Daniel Gyamfi, Yaw Amo Wiafe, Enoch Ofori Awuah, Evans Asamoah Adu, Emmanuel Kodie Boadi

**Affiliations:** ^1^Department of Medical Diagnostics, Faculty of Allied Health Sciences, Kwame Nkrumah University of Science and Technology, Kumasi, Ghana; ^2^Department of Molecular Medicine, School of Medical Sciences, Kwame Nkrumah University of Science and Technology, Kumasi, Ghana

## Abstract

**Background:**

Iodine deficiency is a public health problem. The universal salt iodization (USI) program is the main, simple, and cost-effective intervention strategy to control iodine deficiency. The study examined the iodine status in school-aged children in Ashanti region, Ghana, using thyroid volumes along with urinary iodine concentrations, the methods recommended by the WHO/ICCIDD for monitoring the sustained impact of USI programs.

**Methods:**

This cross-sectional study was conducted among school-aged children (6–12 years) from randomly selected schools in the central and northern part of the Ashanti region, Kumasi Metropolis, and Ejura-Sekyedumase Municipality, respectively. A total of 852 children were enrolled in the study. Thyroid volume and urinary iodine concentrations of the children were determined using the standardized methods recommended by WHO/ICCIDD. Anthropometric measurements were also evaluated.

**Results:**

The mean values of thyroid volume in female and male school-aged children were 3.53 ± 0.09 and 3.32 ± 0.07, respectively. The thyroid size was significantly associated with age (*P* < 0.0001), weight (*P* < 0.0001), height (*P* < 0.0001), BMI (*P* < 0.05), and BSA (*P* < 0.0001) by Pearson's correlation in both males and females. The P50 (median) thyroid volumes of school children investigated in this study were generally larger compared to the WHO/ICCIDD reference data by age and body surface area. The median value of urinary iodine concentration was 201.85 *μ*g/L, which showed significant sex difference (*P* value <0.0001). Excessive iodine nutrition (≥300 *μ*g/L) was observed among 34.4% of male children and 27.6% of female children. Also, 12.8% of the male and 19.5% of the female children had UIC below requirement (<100 *μ*g/L). The criteria of thyroid volume per age yielded a goitre prevalence of 2.2%. In contrast, the criteria of thyroid volume by body surface area yielded a goitre prevalence of 0.9%.

**Conclusion:**

The study clearly indicated that adequate iodine nutrition exists generally among the school children. However, insufficient and excessive iodine intakes still persist among some of the children. The establishment of local reference values for thyroid volume that might be applicable to precisely define goitre prevalence in the Ghanaian context is highly recommended.

## 1. Introduction

Iodine is an essential micronutrient element which is crucial for thyroid function, normal growth, and development [1]. Minute changes in iodine intake are sufficient to reset the thyroid system at different blood thyroid stimulating hormone (TSH) levels [2]. Thus, iodine deficiency from diets and goitrogen intake can result in a variety of health problems collectively known as Iodine Deficiency Disorders (IDDs). These IDDs include goitre, mental retardation, and cretinism as well as infants and young children death [3]. Zimmermann et al. [3] reported that, globally, about two billion individuals have insufficient iodine intake, with those in south Asia and sub-Saharan Africa particularly affected. The social implication of iodine deficiency is its negative effects on children learning ability [4]. As such, school-aged children are deemed the most vulnerable group to iodine deficiency, since growth of tissue and brain development tend to be more uniform during this period [5]. The World Health Organization (WHO) estimated that over 200 million school-aged children in the world are iodine deficient and are at risk of its complications [6]. Also, the International Council for the Control of Iodine Deficiency Disorders (ICCIDD) in an iodine nutrition progress survey reported that Africa is housing about 58 million, the greatest number of iodine-deficient children [7].

Estimates indicate that close to 120,000 children born each year in Ghana are at risk of developing intellectual impairment as a result of iodine deficiency. About 13.0% of these babies have severe impairment in growth and development, resulting in an average of 22-million-dollar loss in productivity each year in Ghana. A major percentage of the affected children grows with diminished intelligence and mental disorders which have many negative consequences on the Ghanaian educational system [8, 9]. Through universal salt iodization (USI), remarkable progress has been made in reducing iodine deficiency in Africa and other parts of the world [10–12]. Ghana adopted this USI program in 1995 and subsequently backed it by a law in 1996 by the Food and Drugs (Amendment) Act 523 [9]. However, a report by the Ghana Health Service in 2014 indicated that IDDs are still high in the country [13]. Other reports available in Ghana indicate that many Ghanaian school-aged children are moderately deficient of iodine [14] and are among the top ten in the world with regard to iodine deficiency [15].

Thyroid volumes and urinary iodine concentrations are important markers for monitoring USI program in a population [16]. Thyroid gland ultrasonography is a more precise method than palpation technique for determining thyroid volume and has been recommended as the standard criterion in epidemiological surveys [17]. Urinary iodine concentration is a well-accepted, cost-effective, and easily obtainable indicator for iodine status [15]. Since a major fraction (over 90%) of iodine absorbed by the body is excreted in the urine, it is considered as a sensitive marker of current iodine intake and can reflect recent changes in iodine status [15]. In order to aid in the monitoring of USI program in Ghana and provide the needed information with regard to iodine nutrition and goitre prevalence in the country, this study examined the iodine status of school-aged children (6–12 years old) in the Ashanti Region, Ghana.

## 2. Methodology

### 2.1. Study Sites

**Figure 1:**
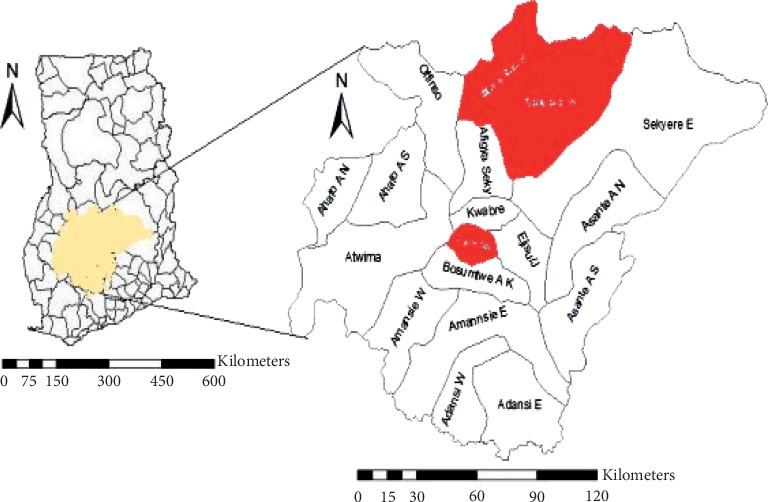
Map of Ashanti region indicating the sampling area (section highlighted in red). Source: adapted from [18].

The research was conducted in four randomly selected schools: Kwame Nkrumah University of Science and Technology (KNUST) Basic School in Kumasi Metropolis, Babaso Municipal Assembly (M/A), and Miminaso M/A and Model M/A Primary Schools in Ejura-Sekyedumase Municipal Assembly, all in Ashanti region of Ghana. KNUST Basic School is located in Kumasi, the capital city of the Ashanti region. It serves a number of suburbs in the city. Ejura-Sekyedumase Municipality is located in the northern part of Ashanti region and northeast of Kumasi. Babaso M/A, Miminaso M/A, and Model M/A Primary Schools are some of the selected schools serving the municipality.  Figure 1 shows the map of Kumasi and the sampling area.

### 2.2. Study Design/Target Population

This is a cross-sectional study that included school-aged children from the ages of 6–12 years. Children who were vegetarians and those who have been restricted of salt intake were excluded. Also, children with history of thyroid surgery were excluded.

### 2.3. Sample Size

To obtain a representative sample size for the entire study population (children within the ages of 6–12 years), at a confidence level of 95% and a margin of error of 0.05, the minimum sample size required for the study was approximately 399 using the formula as follows:

(1) $$\begin{array}{c} n=\frac{N}{1+N{\left(e\right)}^{2}}, \end{array}$$

where *N* is the total school-aged children (255,415) currently attending school in the Ashanti region of Ghana [19], *n* is the estimated sample size, and *e* is the margin of error. However, to ensure a fair distribution of the sample and stronger statistical power and effect, the sample size was projected to 852 children. The study site was divided into two zones which are the Kumasi Metropolis (the central part of Kumasi) and Ejura-Sekyedumase Municipality (the northern zone). A sample of 426 from the central zone and 142 each from the three schools that represented the northern zone in the age group 6–12 years was randomly selected for the study.

### 2.4. Ethical Clearance and Informed Consent

Ethical approval for the study was obtained from the Committee on Human Research, Publication and Ethics, School of Medical Sciences, KNUST, with approval number CHRPE/AP/107/16. Permission was obtained from the schools and Ejura-Sekyedumase Municipality Education Office. Informed consent was obtained from the children's parents or guardians. Consent was administered in English and subsequently translated into the “Ashanti Twi” language. The children verbally gave assent to partake in the study after consent had been obtained from their parents/guardians.

### 2.5. Collection of Demographic, Dietary, and Medical Data

A self-administered questionnaire was used to obtain demographic data. Parents or guardians were made to answer the questionnaire for children below the age of 10 years. The ages of the selected children were determined using records available in the school. Children above 7 years were made to verbally confirm their age. Information on children who were vegetarians, had undergone thyroidectomy, or had been advised to quit salt intake was obtained from the parents and guardians.

### 2.6. Body Composition Measurements

Height was measured to the nearest 0.1 cm using a portable height-rod stadiometer. The weights of the children were also measured using a weighing scale with the reading taken to the nearest 0.1 kg. Both measurements were taken on a flat surface with minimum clothing and emptied pocket and without shoes. A good standing posture was maintained before each measurement was taken. Body surface area (BSA) (m^2^) was calculated by using the Mosteller formula: BSA = [(weight (kg) × height (cm)/3600)]^1/2^ [20]. Body mass index (BMI) was estimated from the ratio of weight (kg) to height (m) square.

### 2.7. Determination of Urinary Iodine Concentration

Guardians or parents of the school-aged children were provided with iodine-free urine containers with screwed caps. Spot morning urine samples were collected from the children and transported on ice to the Micronutrient Research Laboratory at the Cancer and Infection Research Facility, KNUST. The samples were stored at −20°C in Lucky Goldstar (LG, UK) freezer until the day of analysis. Micronutrient Research Laboratory is an active participant of Ensuring the Quality of Urinary Iodine Procedures (EQUIP), an external quality control program established by Centres for Disease Control and Prevention, USA, to ensure good laboratory practice towards the generation of reliable urinary iodine results worldwide [21]. Urinary iodine concentration (UIC) was measured by spectrophotometric method using the Sandell–Kolthoff reaction with ammonium persulfate as the digesting agent. This method is among the three most common methods used by participants of EQUIP program [22]. The intra-assay and interassay coefficients of variation were 4.8–5.8% and 8.9%, respectively. The UIC values were expressed in *μ*g/L, and iodine nutrition classification was defined according to the WHO median urinary iodine level criteria [23]. Categories of median UIC in school-aged children were combined into a single category of “adequate” iodine intake (100 to 299 *μ*g/L) and iodine deficiency (<100 *μ*g/L).

### 2.8. Determination of Thyroid Volume

Thyroid volume was examined for all participants by a single experienced sonographer, using a portable ultrasound machine (Siemens-Acuson P300, Siemens Company, USA), equipped with a 7.5 MHz linear array transducer. During the examination, participants sat straight on a chair with their neck exposed and hyperextended. The echotexture of the thyroid gland was then assessed for signs of nodularity. Afterwards, measurements of the right and left lobes of thyroid gland were obtained from the maximum width, depth, and length, excluding the isthmus. The volume of each lobe was determined as width (cm) × depth (cm) × length (cm) × 0.479. The total volume of the thyroid gland was then calculated as the sum of right and left thyroid lobe volumes. Thyroid gland volumes above the 97^th^ percentile according to the WHO/ICCIDD by age and BSA were accepted as goitre [23, 24]. In case of abnormality in the sonographic examination of the thyroid, the parents of the children received a written note describing the abnormal results of the examination.

### 2.9. Data Analysis

Statistical Package for Social Sciences (SPSS) software version 25 for Windows (Armonk, NY: IBM Corp) was used to analyze the data. Simple descriptive statistics (e.g., frequencies and percentages) and inferences based on chi-square test were performed on categorical variables such as gender, age categories, and iodine concentration categories. Kolmogorov–Smirnov test was used to determine the normality of the distribution of numeric data values. Consequently, nonparametric test (Mann–Whitney *U* test) was utilized to find the association between urinary iodine concentration and gender. Also, the correlation between two continuous variables (urinary iodine concentration and thyroid volume) was performed using Spearman rank correlation. The iodine status of the study population was calculated as median (interquartile range [IQR]). Thyroid volume was represented by mean and standard error. Thyroid volume results were compared with the recommended normal values established by the WHO/ICCIDD [24]. Pearson's correlation and linear regression analysis were done to find the association of age, height, weight, BMI, and BSA with UIC and thyroid volumes. A *P* value less than 0.05 was considered statistically significant.

## 3. Results

The mean age of all the children was 9.8 (0.08 standard error) which did not differ by sex (*P*=0.881) [Table 1]. Male and female children were comparable in terms of height, weight, BMI, and BSA measurements (*P* > 0.05). The mean thyroid volume of the children was 3.43 mL and did not differ significantly by sex (*P*=0.056). The median UIC (201.85 *μ*g/L) was higher in male children compared with female children (224.59 vs 181.02 *μ*g/L, *P* < 0.0001).

**Figure 2:**
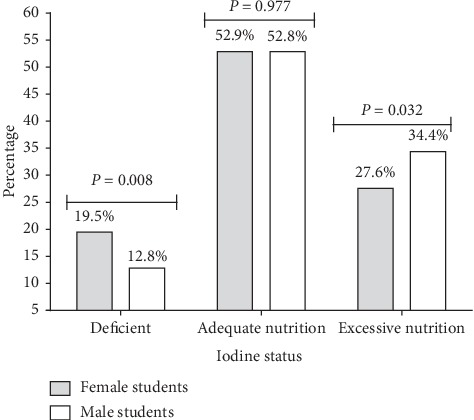
Iodine status of the school-aged children (6–12 years) in the Ashanti region of Ghana stratified by gender.

Iodine status among the females showed that 19.5% were deficient for iodine nutrition, 52.9% had adequate nutrition, and 27.6% had excessive nutrition [Figure 2]. Among the deficient group, the females were significantly higher than the males (*P*=0.008; 19.5% vs 12.8%). Again, the males were significantly higher in proportion within the excessive iodine nutrition group than the females (*P*=0.032; 34.4% vs 27.6%).

In a multiple linear regression, age, height, weight, BMI, and BSA accounted for 40.2% and 1.1% in thyroid volume and UIC among female children, respectively (Table 2). Among male children, these variables accounted for 45.2% and 12.6% variation in thyroid volume and UIC, respectively. Among these predictors, only age significantly predicted thyroid volume (*P* < 0.001) among females and both thyroid volume (*P* < 0.001) and UIC (*P* < 0.001) in males. The highest predictor for thyroid volume in females was weight (*β* = 1.264) and in males was height (*β* = 0.598). The highest predictor for UIC in both males (*β* = −0.373) and females (*β* = −0.747) was BSA.

**Figure 3:**
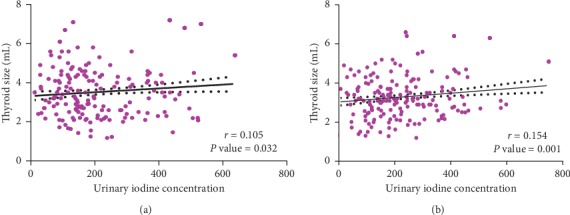
(a) Correlation between urinary iodine concentrations and thyroid volume among female students. (b) Correlation between urinary iodine concentrations and thyroid volume among male students.

**Figure 4:**
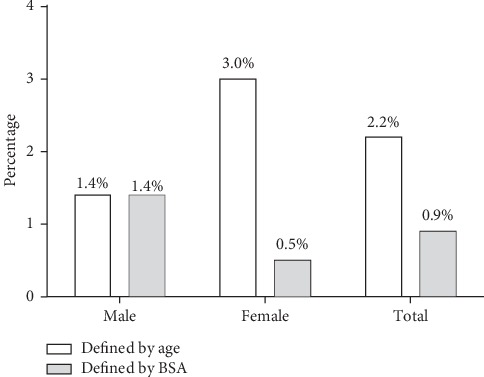
Prevalence of goitre defined by age and BSA among male and female school-aged children (6–12 years) in the Ashanti region of Ghana.

A weak but significant positive correlation (*r* = 0.105, *P* value <0.032; *r* = 0.154, *P* value = 0.001) was observed between urinary iodine concentration and thyroid volumes among males and females, respectively [Figures 3(a) and 3(b)]. The prevalence of goitre defined by age was 3.0% among male children and 1.4% among female children. Also, goitre prevalence by BSA was 1.4% in females and 0.5% among male children. The overall prevalence of goitre by age was 2.2% and 0.9% as defined by BSA [Figure 4].

The descriptive statistics of thyroid volume measured by ultrasonography according to age and BSA is presented in Supplementary Tables 1 and 2. Generally, the median (P50) values and 97th percentile values (P97) were higher among female children compared with the males.

**Figure 5:**
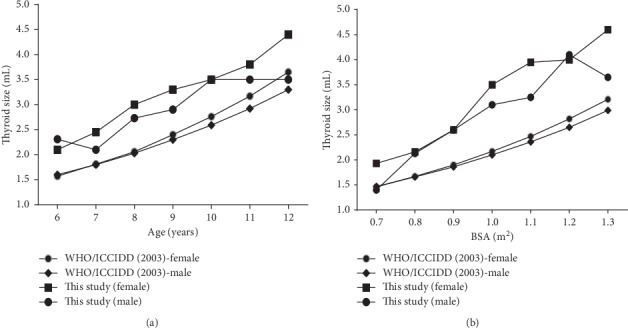
(a) Median (P50) thyroid volume according to age among school-aged children (6–12 years) in the Ashanti region of Ghana, compared with international reference data. (b) Median (P50) thyroid volume according to body surface area (BSA) among school-aged children (6–12 years) in the Ashanti region of Ghana, compared with international reference data.

The comparison of this study to the data from WHO/ICCIDD was performed [Figures 5(a) and 5(b)]. The median thyroid volumes of school-aged children (6–12 years) observed in this study were generally higher than that reported by WHO/ICCIDD (2003) by age and BSA.

**Figure 6:**
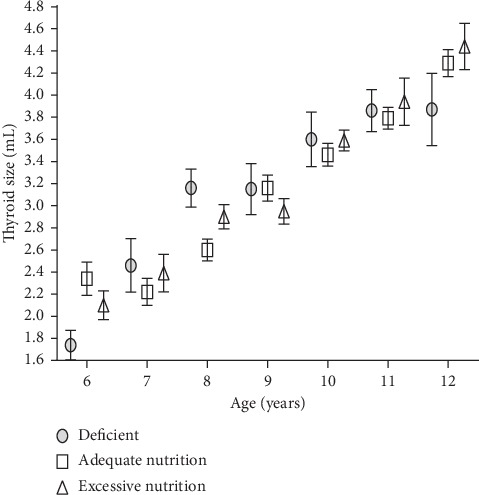
Thyroid volumes in school-aged children with different iodine status. Data are expressed as mean and SEM with whiskers. There was no significant difference in thyroid volumes with respect to iodine status across the various ages (*P* > 0.05).

The mean and standard error estimate for thyroid volumes by age of school-aged children with different iodine status is shown in Figure 6. Mean thyroid volume did not show any significant differences with respect to iodine status across the various ages (*P* > 0.05).

## 4. Discussion

Thyroid ultrasound, along with measurement of urinary iodine levels, has been recommended by WHO/ICCIDD for monitoring the sustained impact of iodine deficiency control programs through universal salt iodization (USI) because it is able to provide information on chronic deficiency of iodine [23]. Our finding provided information on thyroid volumes of school-aged children in a country that has adopted the USI program two decades ago. Although Ghana is located on the iodine deficiency belt, there is lack of evidence on thyroid volume in recent years among school-aged children (6–12 years) since the reports of Amoah et al. [25] in 2004.

Compared with the study of Amoah et al. [25], the mean thyroid volumes in our present findings are lower and did not show any significant difference, although female children had slightly higher volume compared with males. The differences could be attributed to the different age range used; our findings included school children aged 6–12 years compared with 10–15-year age group in their study. One significant reason is that the median urinary iodine concentrations (67.9 *μ*g/L) observed in their study were indicative of iodine-deficient population according to the criteria of WHO/ICCIDD [6]. Comparatively, the median UIC observed in our study (201.85 *μ*g/L) was indicative of adequate iodine intake. Also, goitre prevalence defined by BSA was 0.9% which was lower than 8.0% reported by Amoah et al. [25]. However, goitre prevalence by age observed in this study (2.2%) was similar to that reported by Amoah et al. (1.8%), which did not show significant gender differences. In most iodine-sufficient areas no significant differences in thyroid volumes have been reported between females and males [26], which confirms our present finding. However, the slightly larger thyroid volumes in female school-aged children compared to males could be attributed to the significant differences in the median UIC observed. A significant percentage of females (19.5%) were iodine deficient compared with 12.5% of males. It is important to note that slight alterations in iodine are sufficient to reset the thyroid system at different serum thyroid stimulating hormone (TSH) levels [2].

The results obtained from urinary iodine concentrations in this study indicate that the school-aged children in the Ashanti region of Ghana had adequate iodine with median UIC of 201.85 *μ*g/L. Compared with earlier studies that reported overall low iodine status among school-aged children in Ghana [8, 14], our current finding suggests a massive improvement with regard to iodine nutrition in Ghana, especially the Ashanti region. Since recent amendments in the effective salt iodination strategies by the Ghana Health Service (GHS) in 2014 and the increased educational campaigns by the Ministry of Health in collaboration with GHS on iodine nutrition and iodized salt consumption [13], our finding suggests positive results of this initiative where about 53.0% of male and female school-aged children had adequate nutrition. According to WHO/ICCIDD criteria, median UIC of 100–199 *μ*g/L at the population level indicates adequate iodine intake and optimal nutrition whereas UIC above 300 *μ*g/L is regarded as excessive nutrition [15, 23]. In the present study, 34.4% of male school-aged children and 28.4% of females had a high urinary iodine excretion. This corresponds with a report from Zimbabwe where compulsory iodization program caused median UIC to reach 386 *μ*g/L, indicative of excessive nutrition [27]. Thus, despite milestones reached according to the findings of this study (since the initiative of GHS in collaboration with Ministry of Health), it is recommended that iodine intake should be properly monitored since excess nutrition could result in iodine-induced hyperthyroidism and autoimmune thyroid disease [2].

In our present finding, a multiple linear regression showed that only age significantly predicted thyroid volume. This is consistent with previous reports [26, 28] although the parameter which is more reliable to assess thyroid volume remains to be resolved. In our present study, consistent with several other studies [17, 25, 28], we presented the data on BSA and age-specific thyroid volumes.

In 1999, Langer et al. [29] published a study observing that differences in thyroid volume of siblings are not related to iodine intake, but to other factors including genetic and environmental factors. The bidirectional nature of thyroid volume assessment is that in iodine-deficient areas the effect of iodine deficiency is the most important determinant of thyroid volume, while in an iodine-sufficient area the effects of dietary habits and genetic differences in growth and development are probable indicators of thyroid volume in children [30]. Thyroid volume could not be explained wholly by age, weight, and height which are indicators of growth and development. The value of *R*-square in a multivariate linear regression with age, weight, and height was 0.402. This means that 59.2% of thyroid volume is explained by other factors unaccounted for in this study. In our study, the median thyroid volumes of both male and female school-aged children in the Ashanti region were higher compared to the reference values for sonographic thyroid volume in iodine-replete school-age children data of WHO/ICCIDD [15, 24]. It has been suggested that, because iodine requirement differs by population and geographical area, there is a need for development of population-specific reference range such as that done in Sweden on BSA-specific reference by age [31].

The limitation of the study is that the sample size for some ages or BSA groups was not enough, so the values of 97^th^ percentile for such ages and BSA could not be presented in both male and female school-aged children. The local reference value of thyroid volume establishment is necessary to effectively determine goitre prevalence. Despite this limitation, the study provides important information on thyroid volume and UIC of school-aged children which is a positive directive for USI program in Ghana.

## Figures and Tables

**Table 1:** Descriptive summary of demographic, anthropometric, and biochemical value of participants by sex.

Variable	Total (*n* = 852)	Female children (*n* = 416)	Male children (*n* = 436)	*P* value
Age (years)	9.8 ± 0.08	9.8 ± 0.12	9.8 ± 0.12	0.881
Height (cm)	134.6 ± 0.57	134.9 ± 0.88	134.3 ± 0.76	0.629
Weight (kg)	30.0 ± 0.32	29.8 ± 0.44	30.2 ± 0.45	0.561
BMI (kg/m^2^)	16.4 ± 0.10	16.3 ± 0.15	16.6 ± 0.15	0.206
BSA (m^2^)	1.06 ± 0.01	1.05 ± 0.01	1.06 ± 0.01	0.813
UIC (*μ*g/L)^*∗*^	201.85 (127.43–344.99)	181.02 (118.20–314.77)	224.59 (141.81–367.87)	**<0.0001**
TH VOL (mL)	3.43 ± 0.06	3.53 ± 0.09	3.32 ± 0.07	0.056

BMI: body mass index; BSA: body surface area; TH VOL: thyroid volume; UIC: urinary iodine concentrations. ^*∗*^Values are presented as median (interquartile range). Bold *P* values denote statistically significant correlations.

**Table 2:** Multiple linear regression of age, height, weight, BMI, and BSA with thyroid volume and UIC.

Variable		Female children		Male children	
		TH VOL	UIC	TH VOL	UIC
Model	*R*	0.634	0.107	0.672	0.356
	*R* ^2^	0.402	0.011	0.452	0.126

Constant	*B* (SE)	−1.516 (3.943)	447.870 (545.919)	−7.201 (4.179)	−237.532 (731.547)
	*P* value	0.701	0.412	0.086	0.746

Age	*B* (SE)	0.198 (0.042)	−3.890	0.144 (0.029)	−18.599 (5.132)
	*β*	0.268	−0.049	0.235	−0.219
	*P* value	**<0.001**	0.507	**<0.001**	**<0.001**

Height	*B* (SE)	0.049 (0.077)	0.576 (10.634)	0.056 (0.049)	7.818 (8.640)
	*β*	0.487	0.054	0.598	0.602
	*P* value	0.528	0.957	0.257	0.366

Weight	*B* (SE)	0.0252 (0.274)	7.352	−0.037 (0.130)	13.677 (22.911)
	*β*	1.264	0.343	−0.239	0.633
	*P* value	0.357	0.846	0.774	0.547

BMI	*B* (SE)	−0.011 (0.123)	−8.476	0.155 (0.131)	−3.388 (22.911)
	*β*	−0.018	−0.132	0.335	-0.053
	*P* value	0.931	0.620	0.236	0.883

BSA	*B* (SE)	−10.220 (16.198)	−329.442 (2242.683)	0.130 (7.504)	−693.243 (1313.506)
	*β*	−1.245	−0.373	0.019	−0.747
	*P* value	0.528	0.883	0.986	0.598

BMI: body mass index; BSA: body surface area; TH VOL: thyroid volume; UIC: urinary iodine concentrations. Bold *P* values denote statistically significant prediction.

## Data Availability

The data that support the findings of this study are available from the corresponding author upon reasonable request.

## Supplementary Materials

Supplementary Materials Supplementary Table 1: descriptive statistics of thyroid volume measured by ultrasonography and goitre frequency according to age among male and female students. Supplementary Table 2: descriptive statistics of thyroid volume measured by ultrasonography and goitre frequency according to BSA (m2) among male and female students.
