# Supplementary material for: Goitre Prevalence and Urinary Iodine Concentration in School-Aged Children in the Ashanti Region of Ghana
Source: Int J Endocrinol. 2020 Mar 23;2020:3759786. doi: 10.1155/2020/3759786 (PMC7125487; doi:10.1155/2020/3759786)
Supplement: Supplementary Materials — Supplementary Table 1: descriptive statistics of thyroid volume measured by ultrasonography and goitre frequency according to age among male and female students. Supplementary Table 2: descriptive statistics of thyroid volume measured by ultrasonography and goitre frequency according to BSA (m2) among male and female students. [file 3759786.f1.pdf]

Supplementary Table 1: Descriptive statistics of thyroid volume measured by ultrasonography and Goitre frequency according to age among male and female students

Age (years)	Sex	Students (n)	Minimum (mL)	Maximum (mL)	P50 (mL)	P97 (mL)	Goitre frequency	Goitre prevalence
**6**	Female	22	1.18	3.60	2.10	NC	NC	NC
	Male	26	1.20	3.10	2.31	NC	NC	NC
**7**	Female	26	1.23	3.70	2.45	NC	NC	NC
	Male	26	1.29	3.80	2.10	NC	NC	NC
**8**	Female	58	1.43	4.10	3.00	4.10	4	6.9%
	Male	42	1.31	4.30	2.73	4.30	2	4.8%
**9**	Female	46	2.10	5.40	3.30	5.40	2	4.3%
	Male	74	1.51	5.00	2.90	4.83	2	2.7%
**10**	Female	114	1.67	5.80	3.50	5.40	4	3.5%
	Male	96	1.91	5.20	3.50	5.10	2	2.1%
**11**	Female	72	2.19	7.00	3.80	7.00	1	1.4%
	Male	98	2.13	6.30	3.50	6.30	0	0
**12**	Female	78	2.07	7.20	4.40	7.20	0	0
	Male	74	2.09	6.60	3.50	6.55	2	2.7%

P50 - median; P97 - 97th percentile; NC-not computed

Supplementary Table 2: Descriptive statistics of thyroid volume measured by ultrasonography and Goitre frequency according to BSA (m^2) among male and female students

BSA (m^2)	Sex	Students (n)	Minimum (mL)	Maximum (mL)	P50 (mL)	P97 (mL)	Goitre frequency	Goitre prevalence
**0.7**	Female	4	1.56	2.30	1.93	NC	NC	NC
	Male	10	1.20	2.10	1.40	NC	NC	NC
**0.8**	Female	40	1.18	3.70	2.16	3.70	0	0
	Male	42	1.31	3.50	2.13	3.50	0	0
**0.9**	Female	72	1.23	4.40	2.60	4.38	2	2.8%
	Male	70	1.51	3.96	2.60	3.94	2	2.9%
**1.0**	Female	102	1.67	5.00	3.50	4.90	2	2.0%
	Male	90	2.13	4.70	3.10	4.70	0	0
**1.1**	Female	72	2.10	5.60	3.95	5.54	2	2.8%
	Male	80	2.20	5.20	3.25	5.20	0	0
**1.2**	Female	70	2.50	7.20	4.00	7.20	0	0
	Male	96	2.69	6.40	4.10	6.40	0	0
**1.3**	Female	56	2.10	7.10	4.60	7.10	0	0
	Male	48	2.54	6.60	3.65	6.60	0	0

P50 - median; P97 - 97th percentile; BSA - body surface area; NC-not computed
